# New Insight into Phase Transitions of Porous Glass-Based Ferroelectric Nanocomposites

**DOI:** 10.3390/ma13173698

**Published:** 2020-08-21

**Authors:** Ewa Rysiakiewicz-Pasek, Tatiana Antropova, Irina Polyakova, Olga Pshenko, Agnieszka Ciżman

**Affiliations:** 1 Department of Experimental Phyiscs, Wrocław University of Science and Technology, Wyb. Wyspiańskiego 27, 50-370 Wrocław, Poland; Agnieszka.Cizman@pwr.edu.pl; 2 Laboratory of the Physical Chemistry of Glass, Grebenshchikov Institute of Silicate Chemistry, Russian Academy of Science, Nab.Makarova 2, 199034 Saint Petersburg, Russia; antr2@yandex.ru (T.A.); ira_pp@list.ru (I.P.); zubanova_oa@mail.ru (O.P.)

**Keywords:** nanocomposites, mixed ferroelectrics, structure, electrical conductivity

## Abstract

The results of XRD, FTIR and differential scanning calorimetry (DSC) studies of empty porous silica matrices filled with binary mixtures of K_1–x_Ag_x_NO_3_ (x = 0.05, 0.10) are reported in comparison with those obtained for bulk salts in the temperature range of structural phase transitions. Scanning electron microscopic data of the studied empty macroporous and microporous glasses confirmed differences in the pore morphology associated with the presence of silica gel. Accordingly, XRD and FTIR samples contain crystalline phase of KNO_3_ and AgNO_3_. The results of calorimetric investigation of porous glasses filled with binary mixtures of K_1–x_Ag_x_NO_3_ (x = 0.05, 0.10) are presented. The results show that in the K_1–x_Ag_x_NO_3_ nanocomposites, anomalies associated with phase transitions were detected. An influence of the mean value of pores sizes on the ferroelectric phase transition temperatures of K_1–x_Ag_x_NO_3_ nanocrystals embedded into the porous matrices was determined. The impact of pore space structure on the phase transitions of ferroics nanocomposites was discussed.

## 1. Introduction

Owing to the potentially high dielectric constant and ability to support switchable polarization states and to storing energy, ferroelectric materials are being intensively explored as media for nonvolatile ultra-high-density memories and solar materials. Intense experimental efforts targeted both toward the synthesis of new ferroelectric compounds, as well as the characterization of their physical and materials properties are underway. Molten salt mixtures are in the field of interest of many investigations, owing to their application in chemical industry and energy storage systems [1]. Every bulk A_x_B_1-x_NO_3_ (where A, B = Li, Na, Rb, K, Ca, Cs, Ag; x = 1, 0.75, 0.5, 0.25, 0.1, 0.05) family member has been extensively studied with respect to interesting physical properties [2,3,4,5,6,7]. It was shown that confined geometry has an impact on the structural phase transitions including ferroelectric transitions. The studies of ferroelectric nanocomposites are not numerous, while those materials seem very promising. The most investigated salts ferroelectrics within nanopores are sodium and potassium nitrate, mixtures with sodium nitrate [8,9,10,11,12,13,14]. The results of the investigations of the physical properties of confinement nitrates have been reported [15,16,17,18,19].

The results of dielectric studies of silicate matrices with cellular pores (37.0 and 26.1 Å in diameter) filled by binary mixtures of K_1–x_Ag_x_NO_3_ (x = 0, 0.05, 0.10) have been reported [12,13]. It has been revealed that the permittivity and electrical conductivity increase with increasing AgNO_3_ concentration in bulk samples, as well as with decreasing pore size in the nanocomposites for all values of x. It was also shown that the introduction of small amounts of potassium brings about a noticeable change in the intensity ratio of the elastic Bragg peaks, while leaving the space group characterizing the structure of these nanocomposites unaffected [11]. An increase in the potassium fraction does not result in a substantial decrease in the phase transition point. Measurements of the dielectric response have revealed that an increase in the potassium content gives rise to a marked “hardening” of the lattice in the pre-melting state, which reduces dielectric losses.

The materials such as potassium nitrate (KNO_3_), cesium nitrate (CaNO_3_) and lithium nitrate (LiNO_3_) etc. have been studied in a variety of forms like bulk crystals, opals or thin films in order to use these materials in memory devices operated at low voltage [20,21,22,23]. Because of the high coercive field in bulk materials it is necessary to fabricate them in nanoscale for a low voltage operation. The low voltage, the fast switching time and their great efficiency in storing thermal energy make A_x_B_1-x_NO_3_ nanocomposites promising materials as storage media in large scale integrated access memories (FRAM), switching devices as well as photovoltaic or “rechargeable” batteries. Mixtures of potassium nitrate (KNO_3_) and sodium nitrate (NaNO_3_) are used for storing heat in solar energy installations. This important technological application attracts intense research activity in understanding the properties of the ferroelectric phase in a nanoscale regime. The understanding of the nature of the ferroelectric phase in A_x_B_1-x_NO_3_, is needed, particularly for the possibility of obtaining a stable ferroelectric or superparaelectric phase at room temperatures [9,10]. A determination of the conditions for the photoelectric effect appearance in A_x_B_1-x_NO_3_-porous glass composites requires detailed studies. Moreover, the materials mixed with a small amount of AgNO_3_ compound means that the ferroelectric phase becomes more stable and enhances its optoelectronics properties.

Structural investigations indicate that the bulk potassium nitrate KNO_3_ has the orthorhombic structure Pmnc (phase II) at room temperature [24]. During heating, at temperature 401 K, it transforms to the paraelectric triagonal phase R-3m (phase I). Upon cooling, KNO_3_ can change, before returning to phase II, to the ferroelectric triagonal phase with symmetry R-3m (phase III) at the temperature range 397–373 K. The bulk AgNO_3_ exhibits the orthorhombic structure at room temperature up to 432 K and undergoes to the rhombohedral above this temperature [25].

A synthesis of K_x_Ag_1-x_NO_3_-PG nanocomposites allows us to obtain novel promising materials with properties desired in electronics (for the ferroelectric random-access memory) and optoelectronics (unconventional photovoltaic materials, radiation detectors). PGs-porous glasses, matrices for ferroelectric nanocomposites, possess a complicated structure. So-called microporous glasses exhibit the mesoporous structure as well as microporous substructure while so-called macroporous glasses represent only mesoporous one. A correlation between glass structures (pores morphology or diameter of pores) and phase transitions is interesting from the scientific and technological point of view.

The aim of this work is to study structure and phase transition of binary salts nanocomposites K_1-x_Ag_x_NO_3_ prepared based on porous glasses. According to our knowledge an investigation of structure and thermal properties of porous matrices embedded by K_1-x_Ag_x_NO_3_ mixture has not been conducted up to now.

## 2. Materials and Methods

Porous silicate matrices of two types (MIP—microporous and MAP—macroporous ones) in the form of flat-parallel polished plates with a size of 10 × 10 × 1 mm^3^ were obtained as a result of chemical etching of phase-separated sodium borosilicate (SBS) glass of composition (wt.%): 6.74 Na_2_O, 20.52 B_2_O_3_,·72.59 SiO_2_, and 0.15 Al_2_O_3_ [26] in 3 M HCl solution (glass MIP) and additionally in 0.5 M KOH solution (glass MAP). To form a two-frame structure, SBS glass was previously subjected to isothermal exposure at 550 °C for 144 h. Obtained PGs-porous glasses were washed in distilled water and dried at 120 °C in an air atmosphere. The compositions of PGs and their texture parameters are presented in Table 1 [27,28].

According to the existing generally accepted concepts, the so-called secondary silica is formed in the pore space of the MIP glass during the acid leaching of phase-separated alkaline borosilicate glass with interpenetrating coexisting phases. When etching MIP glass in an alkaline solution, secondary silica is dissolved and extracted from the pore space. As a result, the volume porosity and average pore diameter increase, and the specific pore surface decreases.

To obtain composite materials the MIP and MAP matrices were filled by binary mixtures of K_1–x_Ag_x_NO_3_ (x = 0.05, 0.10) from aqueous salt solution at 80 °C. Three-stage impregnation was performed with intermediate and final drying at 120 °C or 150 °C. As a result the nanocomposite materials PG-MIP05 and PG-MIP1 based on MIP glasses embedded with K_1-x_Ag_x_NO_3_ for x = 0.05 and 0.10 respectively and PG-MAP05 and PG-MAP1 based on MAP glasses for x = 0.05 and 0.10 were obtained.

Structural measurements were performed using the X-ray Diffraction (XRD, “Bourevestnik” Joint-Stock Company, Saint-Petersburg, Russia) and the Fourier-transform infrared (FTIR, “INFRASPEC” Company, Saint-Petersburg, Russia) spectroscopy methods. The results of electron microscopic examination of the PG matrices accordingly [27] were used. SEM analysis was performed using a Zeiss Merlin field emission scanning electron microscope (Carl Zeiss Merlin FESEM, Oberkochen, Germany) with a GEMINI-II electron optics column, and an oil-free vacuum system. The appliance was equipped with additional devices for X-ray microanalysis Oxford Instruments INCAx-act and the backscattered electron diffraction registration system (EBSD) Oxford Instruments CHANNEL5. XRD studies of the composites were performed by X-ray powder diffraction analysis using the modified DRON-2 device (CuK_α_ radiation; tube voltage 38 kV; current 15 mA; the rotation rate of the sample 2 deg/min). Phase analysis was carried out by an electronic search engine PCPDFWIN utilizing a powder diffraction database.

FTIR spectroscopy study of the PG MAP matrix and K_1-x_Ag_x_NO_3_ composites based on it was carried out in the region of 4000–400 cm^−1^ (with a spectral resolution of 4 cm^−1^) using the technique of pressing the samples into tablets with KBr. The spectra were obtained by the spectrophotometer “SPECORD M-80” (Carl Zeiss Jena, Germany). Processing of the data spectra was performed taking into account the atmosphere spectrum and the spectrum from KBr followed by smoothing of the spectral features by the FFT Filter method in accordance with the procedure described in Reference [29].

Differential scanning calorimetry (DSC, Metter Toledo, CO, USA) measurements of obtained nanocomposites were performed on a Mettler Toledo DSC-1 calorimeter at a rate of 2 K min^−1^ under a nitrogen atmosphere with cooling and heating cycles in the temperature range 320–420 K. In the case of porous glasses, samples were additionally annealed in 100 °C for 2 h to remove water.

## 3. Results and Discussion

### 3.1. Structure Characterization

**Figure 1:**
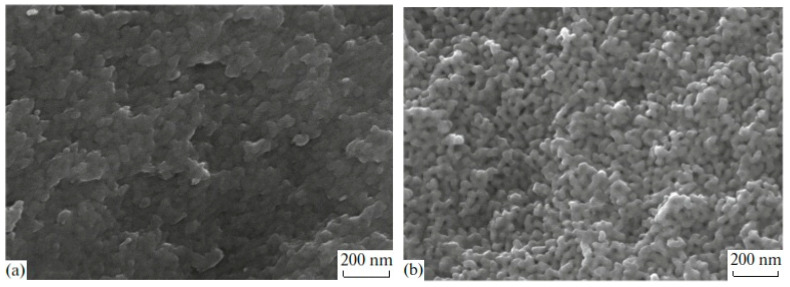
SEM images from cleavages for porous glasses MIP (**a**) and MAP (**b**) [27].

SEM image analysis of MAP and MIP porous glasses have shown that PGs have a branched pore system in the nanometer range (see Figure 1). Due to this, PG matrices have excellent adsorption properties, which allow the introduction of useful substances from aqueous salt solutions into their pore space. The differences in the porous space structure of MIP and MAP glasses are associated with the presence of silica gel inside pores of MIP glass and the lack of it in the MAP glass. This is why the MIP and MAP glasses have different textural pore structure parameters (Table 1), which should affect the amount of substance introduced and, consequently, the properties of the received composite.

**Figure 2:**
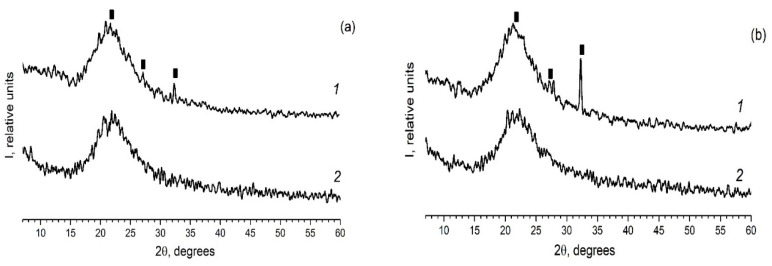
Diffractograms of the composites (**a**) PG-MIP05 and (**b**) PG-MIP1. 1–surface of the sample (plate); 2–volume of the sample (i.e., powder). Black rectangular points refer to the crystalline phase KNO_3_ (3–482).

**Figure 3:**
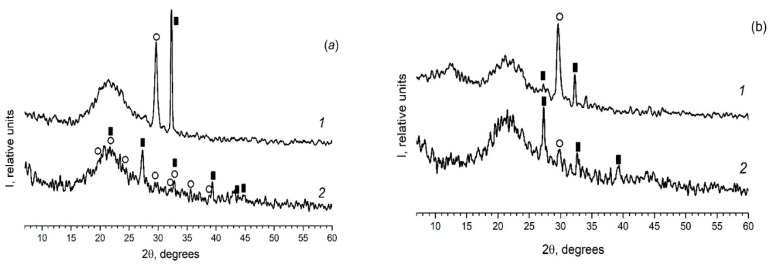
Diffractograms of the composites (**a**) PG-MAP05 and (**b**) PG-MAP1. 1–surface of the sample (plate); 2–volume of the sample (i.e., powder). Black rectangular points refer to the crystalline phase KNO_3_ (3–482), open circle points mark the crystalline phase AgNO_3_ (74–2076).

As in the case of the introduction of the individual ferroelectrics (for example KNO_3_ [30,31]) in the different PG matrices, when using mixed dopants (KNO_3_ + AgNO_3_) the peaks characteristic of the crystalline phases of the dopants are detected on diffractograms of the composites (Figure 2 and Figure 3). Herewith the following influence of the matrix texture parameters (Table 1) is revealed. In the case of thin-pore MIP matrices, only the presence of the crystalline phase of salt, the proportion of which is greater in the mixture (namely, KNO_3_) is detected by the XRD method (Figure 2) and only when shooting from the surface of the sample (data 1 in Figure 2). When using the larger-pored PG MAP matrices, the peaks characteristic of the crystalline phases of both embedded salts (KNO_3_ and AgNO_3_) are noticed (Figure 3). Judging by these diffractograms taken from the sample surface, the increase in ratio of KNO_3_/AgNO_3_ or AgNO_3_/KNO_3_ salts in the mixed dopant (see Figure 3a or Figure 3b, respectively) leads to an increase in the intensity of the main peak of salt, the relative proportion of which in the mixture is more. On diffractograms of the sample volume, this effect of the salt ratio is less pronounced. Such a result may be associated with a small amount of the crystalline phase that is beyond the resolution of the used diffractometer.

**Figure 4:**
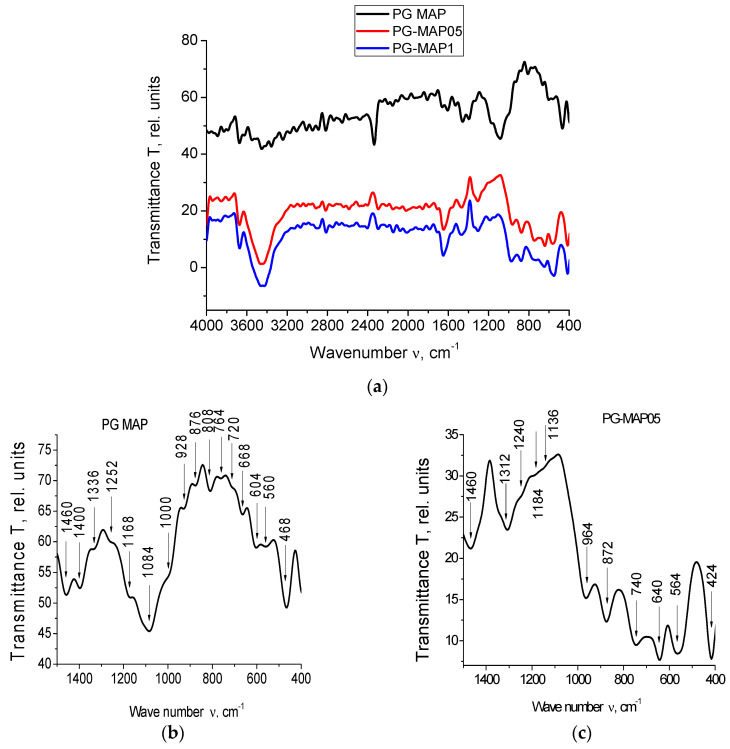

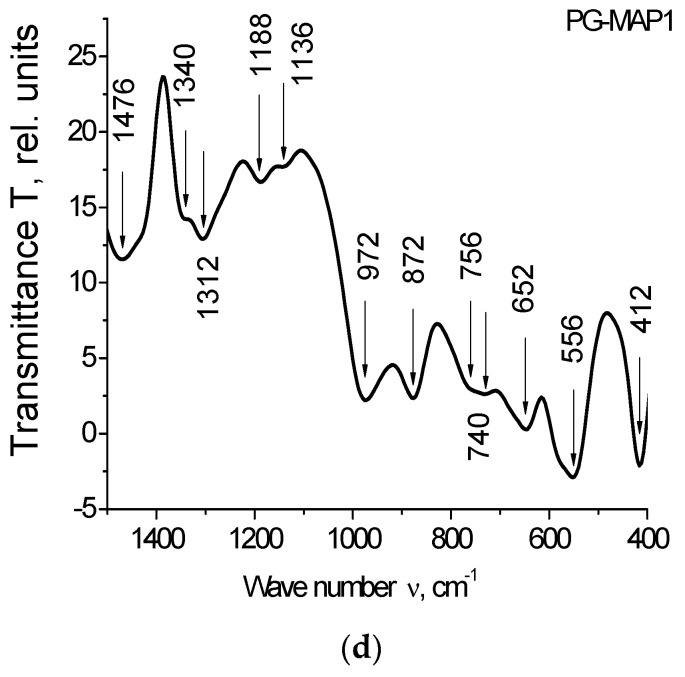
IR spectra of the PG-matrix (PG MAP) and composites (PG-MAP05 and PG-MAP1) in the spectral range (**a**) (4000–400) cm^−1^, (**b**–**d**) (1500–400) cm^−1^.

The IR spectra for PG-MAP-empty porous matrix and composites (PG-MAP05 and PG-MAP1) are presented in Figure 4.

It is seen that the IR spectra of the base matrix (PG-MAP) and of the composites (PG-MAP05 and PG-MAP1) based on it have both similar and different absorption bands. In the case of similar bands, their shift and different intensity can be observed, but the characteristic spectral ranges remain.

According to the literature (see overviews in [32,33,34]) absorption bands in the spectral ranges 3672–3248 cm^−1^ and 2816–2016 cm^−1^ are due to the presence of hydroxyl groups and water in the samples. The bands in the range 1568–1136 cm^−1^ correspond to the vibrations of bonds (B–O) in different boron-oxygen structural groups ([BO_3/2_], [BO_4/2_], etc.). Bands in the ranges 1084–668 cm^−1^ and 564–412 cm^−1^ correspond to vibrations of the (Si–O–Si) bridges and tetrahedra [SiO_4/2_].

Bands at 740 cm^−1^, as well as at 652 cm^−1^ and 640 cm^−1^ are associated with the presence of silver [35,36]. The bands in the range 424–412 cm^−1^ correspond to vibrations of the (K–O) bonds [37].

It should be noted that the bands at 1340/1312 cm^−1^ and 740 cm^−1^ can be attributed to AgNO_3_ (1348 cm^−1^ and 733 cm^−1^ respectively according to [38]). At the same time, taking into account the possible shift, the band at 1340 cm^−1^ can be attributed to KNO_3_ (1380 cm^−1^ according to [38]).

The band at 872 cm^−1^ (taking into account the possible shift) may indicate the presence of both AgNO_3_ and KNO_3_ (835 cm^−1^ and 824 cm^−1^, respectively, according to [38]). The band 1480–1476 cm^−1^ is associated with the presence of anions NO_3_^−^ [39].

### 3.2. Differential Scanning Calorimetry (DSC)

Differential scanning calorimetry (DSC) was used to determine the solid state phase transitions of the salt K_1-x_Ag_x_NO_3_ mixture. In order to improve the temperature resolution of measurements to record the heat flow curve as a function of temperature, a low scanning rate was chosen.

**Figure 5:**
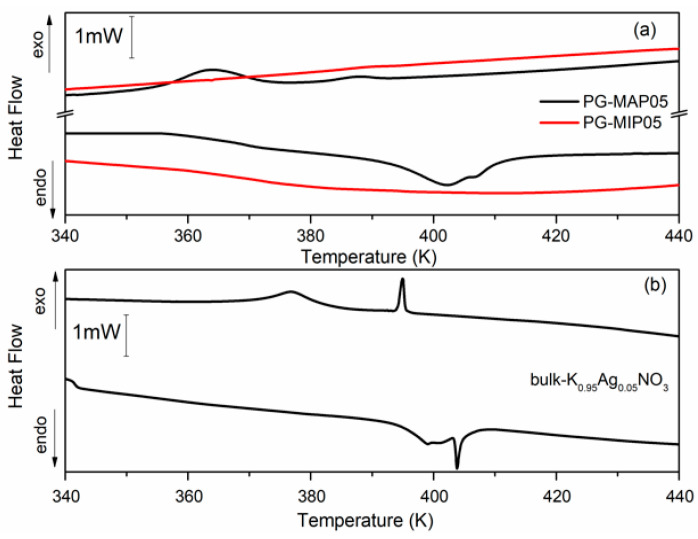
Differential scanning calorimetry (DSC) traces for (**a**) PG-MAP05 and PG-MIP05 nanocomposites and for (**b**) bulk K_0.95_Ag_0.05_NO_3_ during the cooling and heating scans (rate: 2 K min^−1^).

**Figure 6:**
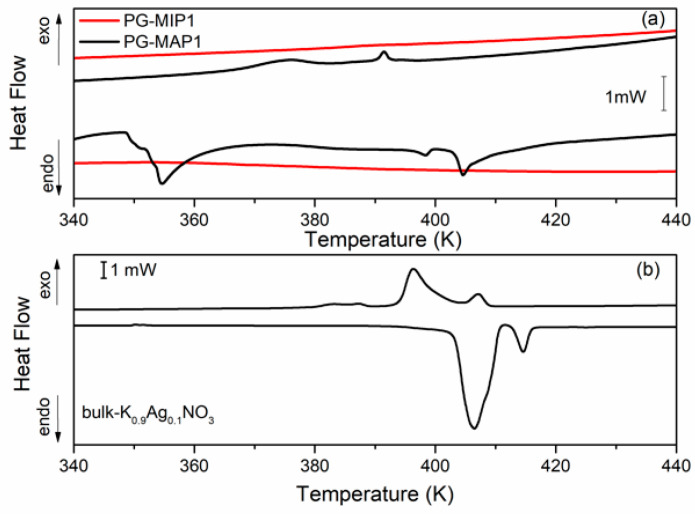
DSC traces for (**a**) PG-MAP1 and PG-MIP1 nanocomposites and for (**b**) bulk K_0.90_Ag_0.10_NO_3_ during the cooling and heating scans (rate: 2 K min^−1^).

Figure 5 and Figure 6 depict the heat flow of bulk and nanocomposites (MIP and MAP) with binary K_1-x_Ag_x_NO_3_ (x = 0.05 and x = 0.10) mixtures vs. temperature. As can been seen (Figure 5b and Figure 6b) for the bulk binary K_1-x_Ag0_x_NO_3_ system during the heating, the anomaly related to the phase transition from II-I phase transition in KNO_3_, is observed at 404 K and 407 K for x = 0.05 and 0.10, respectively. An additional anomaly at 413 K can be related to *α→β* transformation in AgKNO_3_. Investigation of the phase transition in binary bulk K_1-x_Ag_x_NO_3_ for x < 0,4 has been previously performed [40,41]. According to Meszaros Szecesenyi et al. [40] AgNO_3_ undergoes an *α→β* transformation at 432 K for silver nitrate-rich region. Upon the heating phase transitions for composites K_1-x_Ag_x_NO_3_ based on MAP glasses appear at 402 K for x = 0.05 and 404 K for x = 0.10 (Figure 5a and Figure 6a). Probably it is connected with presence of KNO_3_ inside the pores and associated with the phase transition between the phase II and I in the potassium nitrate. The anomaly associated with silver nitrate transformation is invisible.

During the cooling cycle for bulk binary systems with x = 0.05, two anomalies at 376 K and 395 K are revealed. Two anomalies also appear for the bulk binary system with x = 0.10 at 385 K and 397 K (Figure 5b and Figure 6b). These anomalies can be assigned to the I-III and III-II phases seen in KNO_3_ bulk crystals. Similar results were obtained by Meszaros Szecesenyi [40] where the DSC anomaly assigned to the I-III phase transformation in bulk K_1-x_Ag_x_NO_3_ with different AgNO_3_ amount was observed. The third anomaly observed for the bulk binary system with x = 0.10 can be related to the phase transition in AgNO_3_. The anomalies connected with the phase transition between phase I to III and from III to II in KNO_3_ for MAP glasses embedded with K_1-x_Ag_x_NO_3_ occur at 364–389 K for x = 0.05 and 375–392 K for x = 0.10 (Figure 5a and Figure 6a). These phase transition temperatures are lower than in the bulk KNO_3_. The observed tendency of changes of phase transition temperatures in composite materials in comparison with the bulk potassium nitrate is similar to those presented in our previous work [42] and is associated with the size effect. It was seen that for MAP nanocomposites with lower concentrations of silver nitrate the anomalies of heat flow are more smeared. In both MAP05 and MAP1, the observed broadening of phase transitions anomalies can be attributed to the slowing down of the phase transition in restricted geometry [42].

As mentioned earlier, the anomalies associated with phase transition in binary systems have been observed only for MAP nanocomposites, while for MIP binary nanocomposites (Figure 5a and Figure 6a) no visible changes of the specific heat, associated with phase transition, were observed. The absence of a clear maximum on temperatures dependent on the specific heat of MIP nanocomposites may be caused by the presence of silica gel (so-called secondary silica) inside pores of dielectric matrix, which leads to a decrease in the size of the pores that are determined by the size of the gaps between the globules of secondary silica, and have prevented the introduction of the nitrate. According to XRD data, the crystalline phase of KNO_3_ in composites based on PG MIP matrices is detected only in the surface layer of samples (Figure 2, dependencies 1) with extremely low intensity. Judging by the diffractograms of the crushed samples, they are absent or amorphous in volume (Figure 2, dependencies 2), which may be due to a small pore size which does not favor the ferroelectric materials introducing process and thus can determine the critical size of the investigated binary system.

## 4. Conclusions

We report the synthesis, structural properties and phase transitions of K_1-x_Ag_x_NO_3_ porous glasses-based nanocomposites. Based on the results of X-ray diffraction we can conclude that the existence of silica gel inside pores has an impact on porous glass morphology, porosity, specific surface area, and mean diameter of pores. It has been revealed that the intensity of the crystalline phase of KNO_3_ depends on pore size and total ration of KNO_3_/AgNO_3_ in impregnation solution. The influence of AgNO_3_ (x = 0.05 or x = 0.10) content in binary mixtures of K_1-x_Ag_x_NO_3_ on phase transition temperature and the shape of the curve for nanocomposites based on microporous (MIP) and macroporous (MAP) matrices has been observed. It is seen that the structure of pores (MIP or MAP) determines phase transition temperature and the shape of the curve at the same x content.

## Figures and Tables

**Table 1:** Composition and the pore structure parameters of MIP and MAP glasses.

Glass	Composition, As-Analyzed, wt.%					Porosity W, cm^3^/cm^3^ (%)	Specific Surface Area S_A_, m^2^/g	Mean Pore Diameter D, nm
	SiO_2_	Na_2_O	K_2_O	B_2_O_3_	Al_2_O_3_			
MIP	97.11	0.49	0	2.29	0.11	0.29 (29)	164	3
MAP	94.33	0.71	0.59	4.23	0.14	0.57 (57)	87	25
